# *Triplophysa wulongensis*, a new species of cave-dwelling loach (Teleostei, Nemacheilidae) from Chongqing, Southwest China

**DOI:** 10.3897/zookeys.1026.61570

**Published:** 2021-03-26

**Authors:** Shijing Chen, Bakhtiyor Sheraliev, Lu Shu, Zuogang Peng

**Affiliations:** 1 Key Laboratory of Freshwater Fish Reproduction and Development (Ministry of Education), Southwest University School of Life Sciences, Chongqing 400715, China Southwest University Chongqing China; 2 Faculty of Life Sciences, Fergana State University, Fergana 150100, Uzbekistan Fergana State University Fergana Uzbekistan

**Keywords:** Cavefish, *cytb* sequence, freshwater fish, ichthyology, phylogeny

## Abstract

We describe a new species of cave-dwelling loach, *Triplophysa wulongensis***sp. nov.**, based on specimens collected in a subterranean pool in a cave in Wulong County, Chongqing, Southwest China. The pool is connected to the Wujiang River drainage. *Triplophysa wulongensis* differs from its congeners by the following combination of characters: eyes present, caudal fin with 18 branched rays; posterior chamber of the air bladder degenerate; stomach U-shaped; intestine without bends or loops immediately posterior to stomach; body smooth and scaleless, and lateral line complete. The mitochondrial cytochrome *b* sequence differs from those of other published sequences of species of *Triplophysa* by 14.9–24.9% in K2P distance. Phylogenetic analysis based on cytochrome *b* gene sequences recovered *T. wulongensis* as sister taxon to all other cave-dwelling species of *Triplophysa*.

## Introduction

The genus *Triplophysa* Rendahl, 1933, currently comprises approximately 160 valid species, most of which are known from Qinghai-Tibet Plateau and to a lesser extent from Central Asia (Zhu 1989; Prokofiev 2010; Kottelat 2012; Fricke et al. 2020). *Triplophysa* is distinguished from other genera of Nemacheilidae by a marked sexual dimorphism, in which males have tubercle-bearing, elevated skin on the side of the head, and a thickened tuberculated pad on the dorsal surface of the thickened and widened rays of the pectoral fin. Species of *Barbatula* Linck, 1790 share the same sexual dimorphism, but *Triplophysa* can be distinguished from *Barbatula* by the closely situated nostrils (Bănărescu and Nalbant 1968; Prokofiev 2010; Yang et al. 2012; Liu et al. 2017).

To date, 33 cave-dwelling species of *Triplophysa* have been described from the karst areas of southern China where karst caves and subterranean streams are dominant geological features (Lan et al. 2013; Liu et al. 2017; Wu et al. 2018a). According to Lan et al. (2013), these species can be placed into three groups based on the state of the eyes, namely, eyes normal, reduced, or absent (Table 1).

**Table 1.** Characters variable across cave-dwelling species of *Triplophysa* from China.

No	Species	Eyes	Scales	Lateral line	Posterior chamber of air bladder	Dorsal	Anal	Pectoral	Pelvic	Caudal	Tip of pelvic fin reaching anus	Anterior nostril barbel-like
						fin rays	fin rays	fin rays	fin rays	fin rays		
1.	*T. aluensis*	Reduced	Absent	Complete	Degenerated	iii, 7	iii, 5	i, 9	i, 6	13	No	Yes
2.	*T. anshuiensis*	Absent	Absent	Complete	Developed	iv, 7–8	ii, 6	i, 10	i, 6	14	Yes	Yes
3.	*T. baotianensis*	Normal	Absent	Complete	Degenerate	iii, 6–7	ii, 4–5	i, 9	i, 5	11–13	No	Yes
4.	*T. erythraea*	Absent	Absent	Complete	Developed	ii, 8	i, 6	ii, 10	ii, 5	17	Yes	No
5.	*T. fengshanensis*	Absent	Absent	Complete	–	ii, 8	ii, 6	i, 8–10	i, 6–7	16	No	Yes
6.	*T. flavicorpus*	Normal	Present	Complete	Degenerated	iii, 10	iii, 6–7	i, 11	i, 6–7	16	Yes	No
7.	*T. gejiuensis*	Absent	Absent	Complete	Developed	iii, 7–8	iii, 4–6	i, 10	i, 5	14–15	Yes	Yes
8.	*T. guizhouensis*	Normal	Present	Complete	Developed	iii, 8	iii, 6	i, 8–9	i, 6	14	No	Yes
9.	*T. huanjiangensis*	Absent	Absent	Absent	Developed	iii, 8–9	iii, 6–7	i, 10–14	i, 6–7	13–14	No	Yes
10.	*T. huapingensis*	Normal	Present	Complete	Degenerated	iii, 8–9	iii, 5	i, 9–10	i, 5–6	16	No	No
11.	*T. langpingensis*	Reduced	Absent	Incomplete	–	iii, 7–8	iii, 5–6	i, 10–11	i, 6	14	Yes	Yes
12.	*T. lingyunensis*	Reduced	Present	Incomplete	Degenerated	iii, 7–8	iii, 5	i, 8–9	i, 5–6	16	No	Yes
13.	*T. longipectoralis*	Normal	Present	Complete	Degenerated	iii, 8	iii, 5–6	i, 9–10	i, 6	14–15	Yes	Yes
14.	*T. longliensis*	Normal	Absent	Complete	Developed	iii, 8	iii, 5	i, 10	i, 6	15–16	Yes	Yes
15.	*T. luochengensis*	Reduced	Present	Complete	Degenerated	iii, 8	ii, 6	i, 10	i, 6	16–17	No	Yes
16.	*T. macrocephala*	Reduced	Absent	Complete	Degenerated	iii, 7–9	iii, 5–6	i, 9–11	i, 6	15–17	Yes	Yes
17.	*T. maolanensis*	Absent	Absent	Complete	–	iii, 8	ii, 5	i, 11	i, 6	14	Yes	No
18.	*T. nandanensis*	Normal	Present	Complete	Degenerated	iv, 8	iv, 5	i, 9–10	i, 6	14–16	No	Yes
19.	*T. nanpanjiangensis*	Normal	Absent	Complete	Degenerated	iii, 7–8	ii, 5	i, 9–10	i, 6	16	No	Yes
20.	*T. nasobarbatula*	Normal	Present	Complete	Degenerated	iii, 8	iii, 5	i, 9	i, 6	15	Yes	Yes
21.	*T. posterodorsalus*	Absent	Absent	Complete	–	iii, 6	ii, 4	i, 13	i, 5	15	No	Yes
22.	*T. qiubeiensis*	Absent	Absent	Complete	Degenerated	iii, 7	iii, 5	i, 7–9	i, 5	14–15	Yes	No
23.	*T. rosa*	Absent	Absent	Complete	–	iii, 9	iii, 6	i, 12	i, 7	14	Yes	Yes
24.	*T. sanduensis*	Normal	Present	Complete	Degenerated	ii, 8–9	i, 5	i, 8–9	i, 5	17–18	No	Yes
25.	*T. shilinensis*	Absent	Absent	Complete	Degenerated	iii, 7	iii, 5	i, 8–10	i, 6	14	No	Yes
26.	*T. tianeensis*	Reduced	Absent	Complete	Degenerated	iii, 6–7	iii, 5	i, 8–9	i, 5–6	15–16	No	Yes
27.	*T. tianlinensis*	Reduced	Absent	Complete	Degenerated	iii, 7	iii, 5–6	i, 10	i, 6	15–16	Yes	Yes
28.	*T. tianxingensis*	Normal	Absent	Complete	Developed	iii, 8	ii, 5	i, 9	i, 5	16	No	No
29.	*T. wulongensis* sp. nov.	Normal	Absent	Complete	Degenerated	ii, 8–9	i, 5–6	i, 8–9	i, 5–7	18	No	Yes
30.	*T. xiangshuingensis*	Normal	Absent	Complete	Degenerated	iii, 6	iii, 5	i, 9	i, 6	14	No	Yes
31.	*T. xiangxiensis*	Absent	Absent	Complete	Developed	iii, 8	iii, 6	i, 11	i, 6	16	Yes	Yes
32.	*T. xichouensis*	Reduced	Absent	Complete	Developed	iii, 8	ii, 6	i, 9–10	i, 5–6	16	Yes	Yes
33.	*T. yunnanensis*	Normal	Present	Complete	Degenerated	iii, 7	iii, 5	i, 9–10	i, 7	15–16	No	Yes
34.	*T. zhenfengensis*	Normal	Present	Complete	Degenerated	iii, 7	iii, 5	i, 9	i, 5–7	14–15	No	Yes

We collected nine loach specimens from a subterranean pool in a cave located in Wulong County, Chongqing, Southwest China. Morphological and molecular analyses justified the recognition of this sample as representing a new species of *Triplophysa*, described below.

## Materials and methods

After anesthesia, the specimens were fixed in 10% formalin and stored in 70% ethanol. Measurements were made with digital calipers and rounded off to the nearest 0.1 mm. All measurements were made point to point, and whenever possible, measurements and counts were recorded on the left side of the body following the methods described by Kottelat and Freyhof (2007). The standard length was measured from the tip of the snout to the end of the hypural complex; the length of the caudal peduncle was measured from behind the base of the last ray of the anal fin to the end of the hypural complex at mid-height of the base of the caudal fin. The last two branched rays articulating on a single pterygiophore in the dorsal and anal fins were counted as a single ray. Fin rays were counted using a stereo microscope. Vertebrae from five specimens were observed on X-radiographs. The specimens examined were deposited in the Southwest University School of Life Sciences (SWU) in Beibei, Chongqing, P. R. China. Abbreviations are defined as follows: SL, standard length; HL, head length; CLJH, Collection of Lan Jiahu (private collection); GIF, Guangxi Institute of Fisheries, Guangxi, China.

Data on *Triplophysa aluensis* Li & Zhu, 2000, *T. gejiuensis* (Chu & Chen, 1979), *T. nanpanjiangensis* (Zhu & Cao, 1988), *T. qiubeiensis* Li & Yang, 2008, *T. shilinensis* Chen & Yang, 1992, *T. tianxingensis* Yang, Li & Chen, 2016, *T. xiangshuingensis* Li, 2004 and *T. yunnanensis* Yang, 1990 are from Yang et al. (2016); *T. baotianensis* Li, Li, Liu & Li, 2018 and *T. longliensis* Ren, Yang & Chen, 2012 from Li et al. (2018); *T. maolanensis* (Li, Ran & Chen, 2006) and *T. posterodorsalus* (Li, Ran & Chen, 2006) from Li et al. (2006); *T. anshuiensis* Wu, Wei, Lan & Du, 2018, *T. flavicorpus* Yang, Chen & Lan, 2004, *T. guizhouensis* Wu, He, Yang & Du, 2018, *T. luochengensis* Li, Lan, Chen & Du, 2017 and *T. tianlinensis* Li, Li, Lan & Du, 2016 from Wu et al. (2018b); *T. erythraea* Liu & Huang, 2019 and *T. xichouensis* Liu, Pan, Yang & Chen, 2017 from Huang at al. (2019); and *T. xiangxiensis* (Yang, Yuan & Liao, 1986) from Yang et al. (1986). Other species used for comparative purposes were examined at CLJH, GIF, and SWU, China (Suppl. material 1: Table S1).

### DNA extraction and PCR

Genomic DNA was extracted from ethanol-preserved fin tissue using a DNeasy Blood and Tissue Kit (QIAGEN, Shanghai, China). The primers used for PCR amplification of the mitochondrial cytochrome *b* (*cytb*) gene are described by Xiao et al. (2001). PCR amplifications were performed in a total volume of 25 μL consisting of 14.8 μL of dd H_2_O, 2.0 μL of DNA template (50 ng/μL), 1.0 μL of each primer (10 μM), 2.5 μL of 10× PCR buffer, 1.5 μL of 25 mM MgCl_2_, 2.0 μL of 2.5 mM dNTPs, and 0.2 μL of rTaq DNA polymerase (TaKaRa; Dalian, China). The PCR conditions used were as follows: an initial denaturation step at 94 °C for 4 min followed by 34 cycles of 30 s at 94 °C, 50 s at 50–56 °C and 80 s at 72 °C; with a final extension of 8 min at 72 °C.

### Molecular data analyses

We sequenced partial *cytb* gene of *T. longliensis*, *T. nandanensis* Lan, Yang & Chen, 1995, *T. sanduensis* Chen & Peng, 2019, *T. tianeensis* Chen, Cui & Yang, 2004, and *T. wulongensis* and retrieved the *cytb* gene sequences for other species of *Triplophysa* from GenBank (Table 2). *Barbatula nuda* (Bleeker, 1864) and *B. toni* (Dybowski, 1869) were selected as outgroup. Alignment of the *cytb* sequences was performed using the Clustal W algorithm in MEGA7 (Kumar et al. 2016), with manual checks for inconsistencies. MEGA7 was also used to calculate Kimura’s 2-parameter genetic distances (K2P). For phylogenetic reconstructions, the datasets were analyzed based on Bayesian inference (BI) methodology using MrBayes 3.2 (Ronquist et al. 2012) and the maximum likelihood (ML) method of MEGA7 (Kumar et al. 2016). MrBayes used the Generalized Time Reversible model (nst = 6) and gamma-distributed rate variation and the proportion of invariable positions (GTR+G+I) for the *cytb* datasets. For BI, we ran four simultaneous Monte Carlo Markov chains for 2,000,000 generations, with sampling every 1,000 generations, and the first 25% of samples were discarded as burn-in. Tracer v. 1.7 (Rambaut et al. 2018) was used to assess convergence of the posterior, which was determined when effective sample size (ESS) values reached 200. For ML analyses, we conducted heuristic searches (1,000 runs) using a Kimura’s 2-parameter (K2P) model. The phylogenetic trees were visualized and edited using FigTree v. 1.4.2 (Rambaut 2014).

**Table 2.** The species used in this study with their GenBank accession number for the mitochondrial *cytb* gene sequences.

Species	GenBank accession number	Species	GenBank accession number
*Barbatula nuda*	KF574248	*Triplophysa minxianensis*	KT213596
*Barbatula toni*	AB242162	*Triplophysa nandanensis*	MW582824
*Triplophysa anterodorsalis*	KJ739868	*Triplophysa rosa*	JF268621
*Triplophysa bleekeri*	JQ686729	*Triplophysa sanduensis*	MW582822
*Triplophysa brevicauda*	KT213588	*Triplophysa siluroides*	KT213603
*Triplophysa chondrostoma*	KT213589	*Triplophysa tianeensis*	MW582826
*Triplophysa erythraea*	MG967615	*Triplophysa tibetana*	KT224364
*Triplophysa huapingensis*	MG697589	*Triplophysa wulongensis*	MW582823
*Triplophysa lewangensis*	KU987438	*Triplophysa xiangxiensis*	KT751089
*Triplophysa longliensis*	MW582825	*Triplophysa xichangensis*	KT224366
*Triplophysa markehenensis*	KT213594	*Triplophysa zhenfengensis*	MK610360
*Triplophysa microps*	KT213595		

## Results

### Triplophysa wulongensis sp. nov.

Taxon classificationAnimaliaCypriniformesNemacheilidae

13E92C65-3141-5CAA-99E7-2A14E031B734

http://zoobank.org/C5034BEA-EC81-4BC1-ADA8-E45CB1699B46

**Figure 1.**
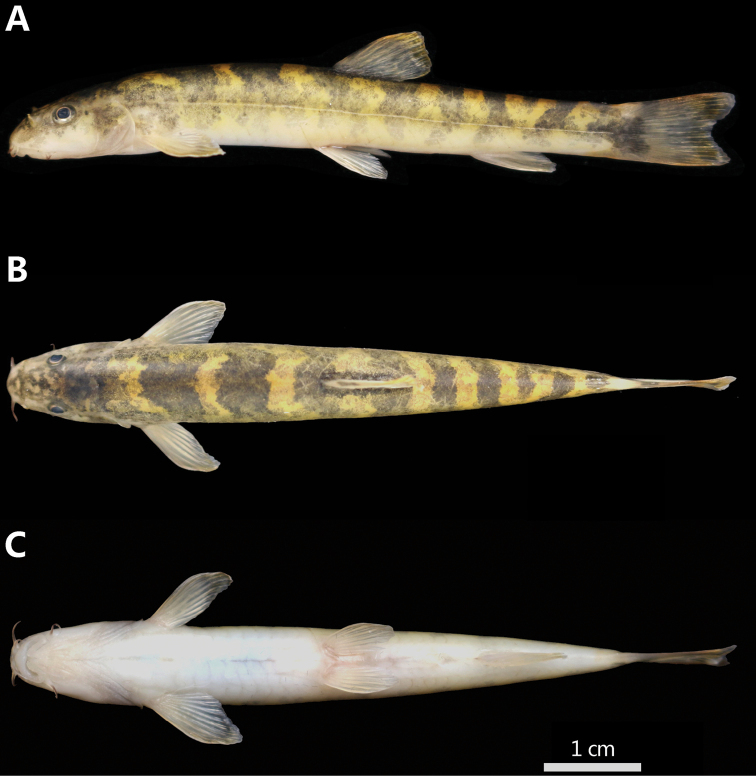
*Triplophysa wulongensis* sp. nov., holotype, SWU 2019051309, 64.0 mm SL**A** lateral view **B** dorsal view **C** ventral view.

**Figure 2.**
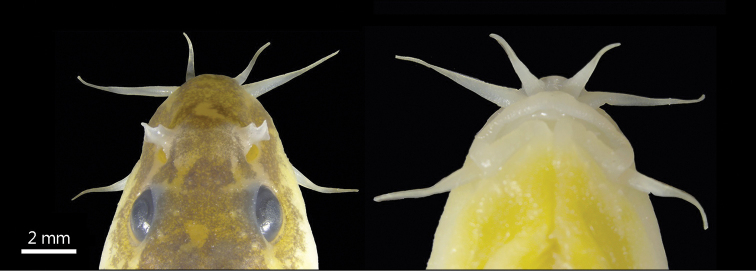
*Triplophysa wulongensis* sp. nov., holotype SWU 2019051309, 64.0 mm SL; head in dorsal and ventral view.

Figures 1 , 2 ; Table 3

#### Type material.

***Holotype*.** SWU2019051309, male, 64.0 mm SL. P.R. China: Chongqing City; Wulong County: subterranean pool in Furong Cave (29°24'1.09"N, 107°54'11.60"E); collected by Ni Liu, May 2019.

***Paratypes*.** SWU2019051301–2019051308, 8 ex., 49.0–67.2 mm SL; collected with the holotype.

#### Diagnosis.

*Triplophysa wulongensis* can be distinguished from its congeners by the following combination of characters: eyes present (vs absent in *T. anshuiensis*, *T. erythraea*, *T. huanjiangensis* Yang, Wu & Lan, 2011, *T. rosa* Chen & Yang, 2005, *T. xiangxiensis* and *T. posterodorsalus*); anterior nostril barbel-like (vs anterior nostril not elongate to barbel-like in *T. erythraea*, *T. flavicorpus*, *T. huapingensis* Zheng, Yang & Chen, 2012 and *T. tianxingensis*): caudal fin with 18 branched rays (vs 14–16 in *T. guizhouensis*, *T. lingyunensis* (Liao, Wang & Luo, 1997), *T. nandanensis*, *T. shilinensis* and *T. zhenfengensis* Wang & Li, 2001), vertebrae 4+38–39 (vs 36–37 in *T. nasobarbatula* Wang & Li, 2001 and *T. sanduensis*; 42–43 in *T. siluroides*); predorsal length 50.4–54.2% of standard length (vs 46.1–48.0% in *T. sanduensis*); posterior chamber of gas bladder degenerate (vs developed in *T. anshuiensis*, *T. tianxingensis* and *T. xichouensis*); body smooth and scaleless (vs body covered by scales in *T. longipectoralis* Zheng, Du, Chen & Yang, 2009 and *T. yunnanensis*); lateral line complete (vs incomplete in *T. huanjiangensis*); and pelvic-fin tip not reaching to anus (vs reaching to anus in *T. gejiuensis*, *T. macrocephala* Yang, Wu & Yang, 2012, *T. rosa* and *T. qiubeiensis*).

#### Description.

Morphometric data of the type specimens of *T. wulongensis* are presented in Table 3. D, 2/8–9; A, 1/5–6; P, 1/8–9; V, 1/5–7; C, 18; vertebrae: 4+38–39 (five specimens).

**Table 3.** Morphometric data of type specimens of *Triplophysa wulongensis* sp. nov. SD = standard deviation.

Morphometric characters	Holotype	Paratypes (SWU2019051301–08)			
	SWU2019051309	Min	Max	Mean	SD
SL (mm)	64	49	67.2	55.7	
% SL					
Lateral head length (HL)	22.9	20.4	23.5	22.6	1
Body depth	13.3	9.3	13.6	12.1	1.3
Predorsal length	54.2	50.4	53.3	51.9	0.9
Postdorsal length	40.7	34	39.4	36.7	1.4
Prepelvic length	49.6	48.3	50.9	49.7	0.9
Preanal length	76	71.5	77.7	73.4	1.8
Preanus length	70	67.4	70.2	69	1
Dorsal-fin height	15.9	15	19.8	16.5	1.5
Dorsal-fin base length	12	10.7	13.4	12.4	0.9
Anal-fin height	14.1	12.4	16.5	14.5	1.3
Anal-fin base length	6.6	6.6	8.2	7.4	0.6
Pelvic-fin length	12.4	12.5	14.5	13.2	0.6
Pectoral-fin length	16.6	15.6	18.4	17.6	1
Caudal-fin length	17.8	15.9	20.8	18.1	1.3
Caudal-peduncle length (CPL)	20.8	14.2	18.4	16.6	1.2
Caudal-peduncle depth (CPD)	9.4	7.6	9.4	8.5	0.7
Pectoral-pelvic distance	26.8	24.6	28.6	26.6	1.1
Pelvic-anal distance	26.4	21.6	26.9	23.7	1.4
Vent-anal fin origin distance	6.2	4	7.2	5.8	1
%HL					
Head depth	53.8	45.3	54.2	50.6	2.9
Head width	62.2	55.7	65.8	62.4	3.5
Snout length	39	38.9	45	41.9	1.9
Eye diameter	11.1	12.2	19.1	17	2.1
Interorbital width	38.7	38.5	43.1	41.3	1.5
Postorbital head length	45	37.9	46.8	43.8	2.8
Maxillary barbel length	21.8	27.2	35.9	29.8	3.2
Inner rostral barbel length	16.5	20.1	23.4	21.6	1.2
Outer rostral barbel length	21.4	25.9	41.5	32.4	4.5
CPD/CPL	45.3	44.3	57.4	51.2	4.5

Body elongated, slightly compressed anteriorly and more strongly compressed posteriorly. Deepest point of body in front of dorsal fin origin, body depth 9.3–13.6% of SL. Caudal peduncle depth/caudal peduncle length range from 44.3% to 57.4%. Head depressed, width greater than depth (62.4% vs 50.6% of HL). Snout moderately blunt and snout length almost equal to postorbital length, approximately 38.9–45.0% of HL. Anterior and posterior nostrils adjacently located; anterior nostril in short tube, each with tip elongated to form a short barbel. Tip of nostril appendage not reaching the anterior margin of eyes. Eyes present, diameter 11.1–19.1% of HL. Mouth inferior, arched; mouth corner situated below anterior nostril. Lips thin; lower lip with well-marked, V-shaped, median notch (Fig. 2). Upper jaw covered by upper lip; lower jaw scoop-shaped, not covered medially by lower lip. Three pairs of barbels; inner rostral barbel extending to rictus, 16.5–23.4% of HL; outer rostral barbel not extending to anterior margin of eyes, 21.4–41.5% of HL; maxillary barbel extending to anterior margin of eyes, 21.8–35.9% of HL.

Dorsal fin emarginate, origin posterior to pelvic fin insertion, situated slightly posterior to midpoint between snout tip and caudal fin base; first branched ray longest; dorsal fin height shorter than lateral head length; tip of dorsal fin reaching vertical of anus. Pectoral fin moderately developed, 56.6–72.9% of distance between pectoral fin and pelvic-fin origins. Pelvic-fin origin situated almost at midpoint between pectoral-fin origin and anal-fin origin, tip of pelvic fin not reaching to anus. Anal-fin origin situated almost at midpoint between pelvic-fin origin and caudal-fin base, distal margin of anal fin truncate; posterior tip of anal fin reaching approximately half distance between anal-fin origin and caudal-fin base. Vent-anal fin-origin distance 4.0–7.2% of SL. Caudal fin emarginate.

Body smooth and scaleless. Cephalic lateral line system developed. Lateral line complete, ending at caudal-fin base. Intestine without bends or loops immediately posterior to stomach; stomach U-shaped. Posterior chamber of gas bladder degenerate.

#### Coloration.

In formalin-fixed specimens, body yellowish dorsally, gradually lighter toward ventral side. Fins semitransparent. Body dorsally and laterally covered with irregular, brown blotches; 6–8 distinct dark brown blotches along dorsal midline.

#### Sexual dimorphism.

Sexual dimorphism was not detected. This may reflect that the sampling time was outside the breeding season of this species.

#### Geographical distribution.

**Figure 3.**
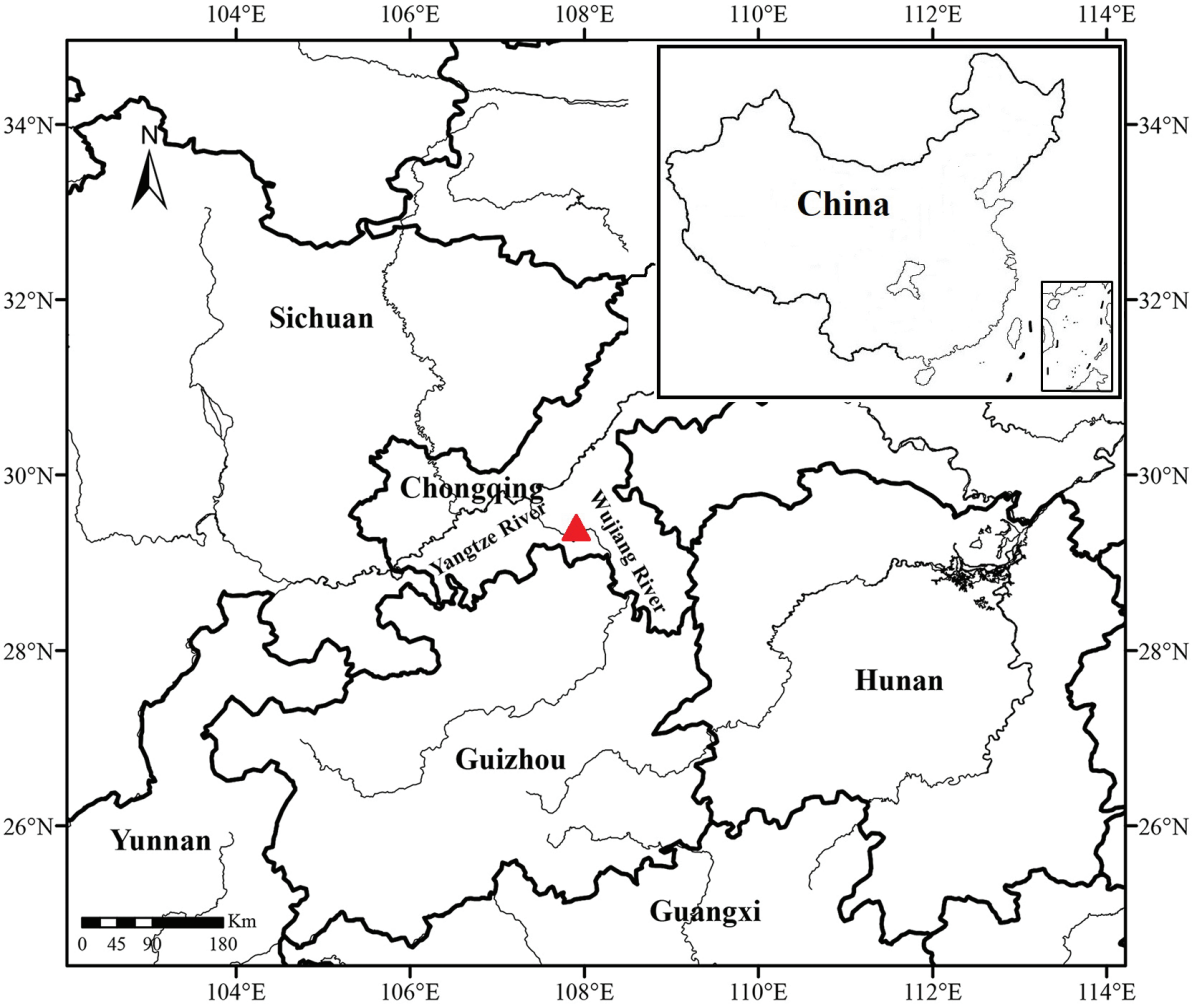
Collection site of *Triplophysa wulongensis* sp. nov. (red triangle) in Chongqing, Southwest China.

Known only from the type series, from a pool in Furong Cave, connected to the Wujiang River near Wulong, (Fig. 3). *Triplophysa wulongensis* was found syntopic with *T. rosa*.

#### Etymology.

The specific name, *wulongensis*, refers to the type locality in Wulong County, where the type specimens were collected; it is an adjective with alternative endings -*is* and -*e*.

## Discussion

**Figure 4.**
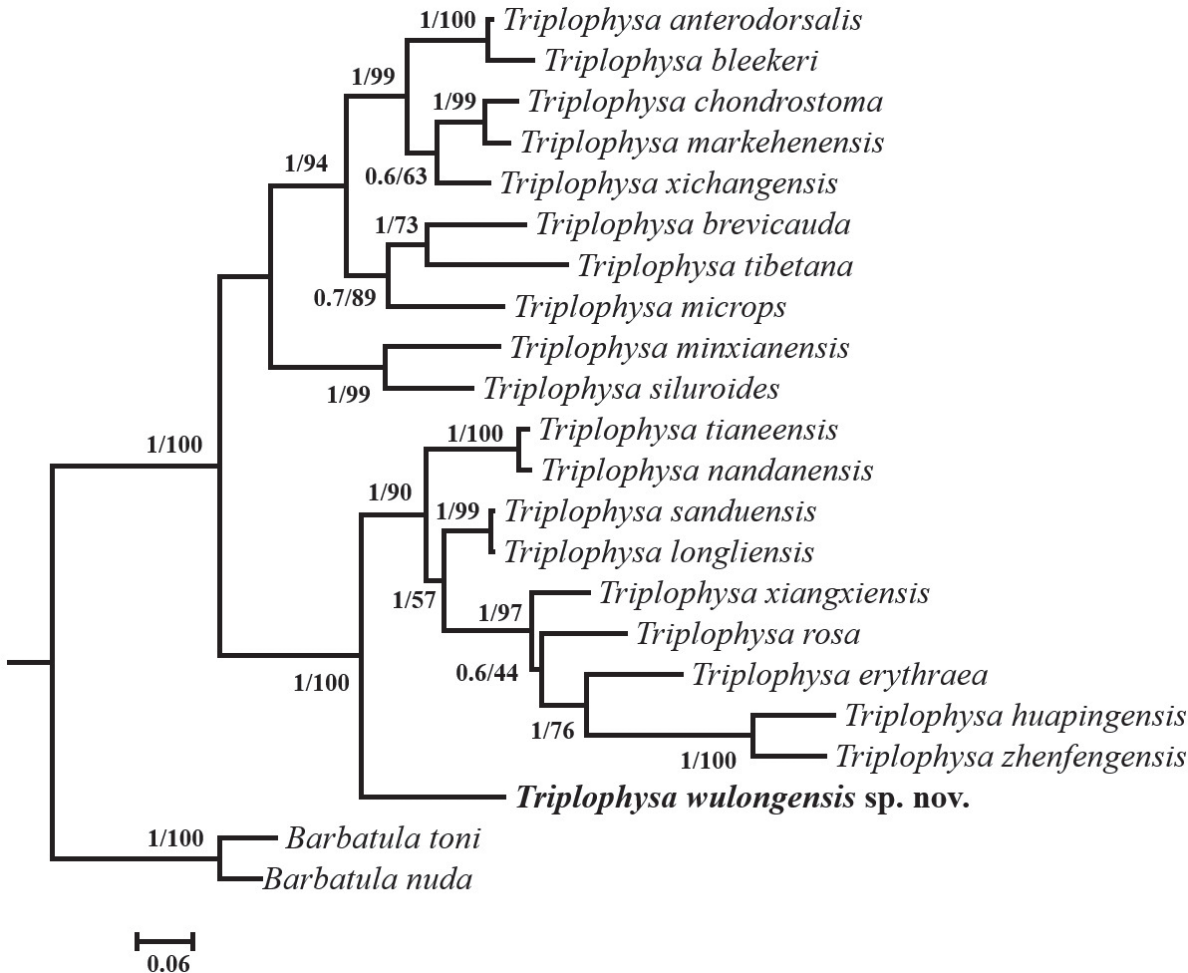
Phylogeny of some species of *Triplophysa* and two outgroup species based on maximum likelihood (ML) and Bayesian inference (BI) methods using mitochondrial *cytb* gene sequences. The ML bootstrap values and BI posterior probabilities are shown at the nodes

In previous studies, the cave dwelling species of *Triplophysa* were nested in a basal position to congeners in phylogenetic reconstructions (Wang et al. 2016; Chen and Peng 2019; Wu et al. 2020). Our phylogenetic analysis based on *cytb* (Fig. 4) resolved two monophyletic clades, one of which comprises cave-dwelling species, and the other includes non-cave-dwelling species, concordant with Chen and Peng (2019). *Triplophysa wulongensis* is located in a basal position of the cave-dwelling clade (Fig. 4). The K2P genetic distances show less differentiation between *T. wulongensis* and *T. sanduensis* (14.9%) than between *T. wulongensis* and its other congeners in this study. The K2P genetic distance (ranges from 14.9% to 24.9%) between the new species and some of the other species of *Triplophysa* based on *cytb* markers is consistent with species-level divergences in other fish taxa (Ward et al. 2005; Wang et al. 2016; Wu et al. 2020).

The presence or absence of the secondary sexual characteristics is important for the generic diagnosis of loaches (Bănărescu and Nalbant 1968; Zhu 1989). The presence of tubercles on the pectoral fin can be considered as an autapomorphy and is the single diagnostic character of *Triplophysa* (Prokofiev 2010). Nevertheless, according to Liang and Zhou (2019), some cave-dwelling species of *Triplophysa*, e.g. *T. nasobarbatula* and *T. zhenfengensis*, have lost secondary sexual characteristics. Sexual dimorphism was not evident in the type series of *T. wulongensis*, but the phylogenetic analysis confirmed the generic classification.

The majority of the cave-dwelling species of *Triplophysa* were described from karst caves and subterranean streams in the Pearl river basin and the upper Yangtze river basin, with an additional two species (*T. erythraea* and *T. xiangxiensis*) reported from the Yuanjiang river drainage (a tributary of the middle Yangtze River) and a single species (*T. rosa*) described from the Wujiang river drainage (Lan et al. 2013; Liu et al. 2017; Wu et al. 2018b; Chen and Peng 2019; Huang et al. 2019). In terms of morphology, *T. wulongensis* is distinguished from the co-occurring *T. rosa* by the presence of eyes (vs absence), 8 or 9 branched pectoral-fin rays (vs 12), 18 branched caudal-fin rays (vs 14), a pectoral fin length that is 15.6–18.4% that of the SL (vs 26.6%), and a body with irregular brown blotches (vs pale blotches).

The rate of discovery of new cave-dwelling species of *Triplophysa* has increased in recent years (Yang et al. 2016; Li et al. 2017a, b, 2018; Liu et al. 2017; Wu et al. 2018a, b; Chen and Peng 2019; Huang et al. 2019), while a taxonomic revision of these species is lacking. Hence, further systematic and phylogenetic study based on both morphometric and molecular approaches is needed.

### Key to the cave-dwelling species of *Triplophysa*

**Table:** 

1	Eyes normal	**2**
–	Eyes reduced or absent	**16**
2	Scales absent	**3**
–	Body covered by scales	**8**
3	Tip of pelvic fin reaching anus, outer gill rakers on first gill arch absent	***T. longliensis***
–	Tip of pelvic fin not reaching anus; outer gill rakers on first gill arch present	**4**
4	Posterior chamber of air bladder developed; anterior nostril not elongate to barbel-like	***T. tianxingensis***
–	Posterior chamber of air bladder degenerated; anterior nostril elongate to barbel-like	**5**
5	Dorsal-fin origin closer to caudal-fin base than to snout tip	***T. wulongensis* sp. nov.**
–	Dorsal-fin origin closer to snout tip than to caudal-fin base	**6**
6	Dorsal-fin origin opposite vertical line trough pelvic-fin origin	***T. nanpanjiangensis***
–	Dorsal-fin origin anterior to vertical line trough pelvic fin origin	**7**
7	Caudal fin deep forked with 11–13 branched fin rays	***T. baotianensis***
–	Caudal fin slightly forked with 14 branched fin rays	***T. xiangshuingensis***
8	Processus dentiformis present in upper jaw	***T. zhenfengensis***
–	Processus dentiformis absent in upper jaw	**9**
9	Posterior chamber of air bladder developed	***T. guizhouensis***
–	Posterior chamber of air bladder degenerated	**10**
10	Tip of depressed pelvic fin exceeding anus	**11**
–	Tip of depressed pelvic fin not reaching anus	**13**
11	Anterior nostril not elongate to barbel-like; branched dorsal-fin rays 10	***T. flavicorpus***
–	Anterior nostril elongate to barbel-like; branched dorsal-fin rays 8	**12**
12	Tip of pectoral fin extending beyond pelvic-fin origin	***T. longipectoralis***
–	Tip of pectoral fin not reaching pelvic-fin origin	***T. nasobarbatula***
13	Branched dorsal-fin rays 7, branched anal-fin rays 7	***T. yunnanensis***
–	Branched dorsal-fin rays 8, branched anal-fin rays 5–6	**14**
14	Dorsal-fin origin opposite vertical line trough pelvic-fin origin	***T. nandanensis***
–	Dorsal fin origin anterior to vertical line trough pelvic fin origin	**15**
15	Anterior nostril not elongate to barbel-like; branched caudal-fin rays 16	***T. huapingensis***
–	Anterior nostril elongate to barbel-like; branched dorsal-fin rays 17–18	***T. sanduensis***
16	Eyes reduced	**17**
–	Eyes absent	**24**
17	Body covered with scales	**18**
–	Scales absent, body smooth	**19**
18	Lateral line complete, branched anal-fin rays 6	***T. luochengensis***
–	Lateral line incomplete, branched anal-fin rays 5	***T. lingyunensis***
19	Lateral line incomplete; adipose keels present on upper or lower side of caudal peduncle	***T. langpingensis***
–	Lateral line complete; adipose keels absent from caudal peduncle	**20**
20	Posterior chamber of air bladder developed	***T. xichouensis***
–	Posterior chamber of air bladder degenerated	**21**
21	Tip of pelvic fin reaching anus	**22**
–	Tip of pelvic fin not reaching to anus	**23**
22	Tip of pectoral fin reaching to midway between pectoral-fin origin and pelvic-fin origin; Spots absent from body	***T. tianlinensis***
–	Tip of pectoral fin reaching a vertical through dorsal-fin origin; spots present on body	***T. macrocephala***
23	Dorsal-fin origin posterior to or at to vertical line trough pelvic-fin origin; branched caudal-fin rays 13	***T. aluensis***
–	Dorsal-fin origin anterior to vertical line trough pelvic-fin origin; branched caudal-fin rays 15–16	***T. tianeensis***
24	Lateral line absent	***T. huanjiangensis***
–	Lateral line complete	**25**
25	Tip of pelvic-fin not reaching to anus	**26**
–	Tip of pelvic fin reaching to anus	**28**
26	Adipose keels present on upper or lower side of caudal peduncle	***T. posterodorsalus***
–	Adipose keels absent from caudal peduncle	**27**
27	Branched dorsal-fin rays 8; branched caudal-fin rays 16	***T. fengshanensis***
–	Branched dorsal-fin rays 7; branched caudal-fin rays 14	***T. shilinensis***
28	Anterior nostril not elongate to barbel-like	**29**
–	Anterior nostril elongate to barbel-like	**31**
29	Lips developed, papillary process absent, branched caudal-fin rays 17	***T. erythraea***
–	Lips developed, papillary process present, branched caudal-fin rays 14–15	**30**
30	Branched dorsal fin rays 8; branched pectoral-fin rays 11	***T. maolanensis***
–	Branched dorsal-fin rays 7; branched pectoral-fin rays 7–9	***T. qiubeiensis***
31	Distal margin of dorsal fin truncate; branched dorsal-fin rays 7–8; branched pectoral-fin rays 9–11; branched pelvic-fin rays 6	**32**
–	Distal margin of dorsal-fin concave; branched dorsal-fin rays 9; branched pectoral-fin rays 12; branched pelvic-fin rays 7	***T. rosa***
32	Snout blunt; tip of pectoral fin not reaching vertical level of dorsal fin origin; tip of caudal-fin lobes pointed; branched caudal-fin rays 14–15	**33**
–	Snout rectangle-like; tip of pectoral fin reaching a vertical through dorsal-fin origin; tip of caudal-fin lobe sharp; branched caudal-fin rays 16	***T. xiangxiensis***
33	Cephalic lateral-line canals with 5 supraorbital and 7 preoperculo-mandibular pores	***T. gejiuensis***
–	Cephalic lateral-line canals with 8 supraorbital and 12–13 preoperculo-mandibular pores	***T. anshuiensis***

## Supplementary Material

XML Treatment for Triplophysa wulongensis

## Supplementary materials

Supplementary material 1 Table S1 Data type Specimen list Explanation note Material examined of *Triplophysa* species from China. https://binary.pensoft.net/file/524712This dataset is made available under the Open Database License (http://opendatacommons.org/licenses/odbl/1.0/). The Open Database License (ODbL) is a license agreement intended to allow users to freely share, modify, and use this Dataset while maintaining this same freedom for others, provided that the original source and author(s) are credited.Shijing Chen, Bakhtiyor Sheraliev, Lu Shu, Zuogang Peng
