# Supplementary material for: Triplophysa wulongensis, a new species of cave-dwelling loach (Teleostei, Nemacheilidae) from Chongqing, Southwest China
Source: Zookeys. 2021 Mar 26;1026:179–92. doi: 10.3897/zookeys.1026.61570 (PMC8018939; doi:10.3897/zookeys.1026.61570)
Supplement: Supplementary material 1 — Table S1 [file zookeys-1026-179-s001.docx]

**Supplementary material 1**

**Table S1. Material examined of *Triplophysa* species from China**

Authors: Shijing Chen, Bakhtiyor Sheraliev, Lu Shu, Zuogang Peng

Data type: Specimen list

Copyright notice: This dataset is made available under the Open Database License (http://opendatacommons.org/licenses/odbl/1.0/). The Open Database License (ODbL) is a license agreement intended to allow users to freely share, modify, and use this Dataset while maintaining this same freedom for others, provided that the original source and author(s) are credited.

**Table S1. Material examined of *Triplophysa* species from China**

| Species | Catalog Number | Number of  Specimens | Standard  Length (mm) | Locality |
| --- | --- | --- | --- | --- |
| *T. fengshanensis* | CLJH 2010110506-07, 2011120701-03 | 5 | 37.2-77.5 | Fengshan, Guangxi |
| *T. huanjiangensis* | GIF 07040316; CLJH 07040308, 07040311, 07040314, 07040319 | 5 | 47.8-122.4 | Huanjiang, Guangxi |
| *T. huapingensis* | CLJH 2008110030-31, 2008110033-37 | 7 | 40.0-75.2 | Leye, Guangxi |
| *T. langpingensis* | CLJH 12120309-10, 2011090398, 201110402-04 | 6 | 36.0-74.9 | Langping, Guangxi |
| *T. lingyunensis* | CLJH 02050589-90, 02050592-93, 10120603 | 5 | 42.6-53.9 | Lingyun, Guangxi |
| *T. longipectoralis* | CLJH 01050216, 01050222, 04050261, 04050265-66, 04050301-02 | 7 | 47.9-80.2 | Huanjiang, Guangxi |
| *T. macrocephala* | CLJH 04100611-12, 04100614-17, 04100620, 04100623, 04100626-30, 04100632, 12030113 | 15 | 40.3-91.4 | Nandan, Guangxi |
| *T. nandanensis* | CLJH 91098662, 91098665-69, 91098671, 91098675-77, 91098679-80, 01080635, 01090637, 02040089 | 15 | 47.2-82.1 | Nandan, Guangxi |
| *T. nasobarbatula* | SWU 20180510002-04 | 3 | 50.1-61.8 | Libo, Guizhou |
| *T. rosa* | SWU 20131114007-18 | 12 | 40.5-105.6 | Wulong, Chongqing |
| *T. sanduensis* | SWU 2017061301-06 | 6 | 45.2-78.8 | Sandu, Guizhou |
| *T. tianeensis* | CLJH 02121521, 02121524-25, 02121527, 02121535-37, 02121539-43, 02121545-46 | 14 | 47.6-67.6 | Tian’e, Guangxi |
| *T. zhenfengensis* | SWU 20160905002-04 | 3 | 52.0-59.1 | Zhenfeng, Guizhou |
